# Genetic Diversity Assessment of MPOB-Senegal Oil Palm Germplasm Using Microsatellite Markers

**DOI:** 10.1155/2021/6620645

**Published:** 2021-05-04

**Authors:** Khin Aye Myint, Zulkifli Yaakub, Mohd Y. Rafii, Yusuff Oladosu, Mohd Yusoff Abd Samad, Shairul Izan Ramlee, Suzana Mustaffa, Fatai Arolu, Norziha Abdullah, Marhalil Marjuni, Mohd Din Amiruddin

**Affiliations:** ^1^Laboratory of Climate-Smart Food Crop Production, Institute of Tropical Agriculture and Food Security, University Putra Malaysia, 43400 UPM Serdang, Selangor, Malaysia; ^2^Advanced Biotechnology and Breeding Centre, Malaysian Palm Oil Board, 6, Persiaran Institusi, Bandar Baru Bangi, 43000 Kajang, Selangor, Malaysia; ^3^Department of Soil Management, Faculty of Agriculture, Universiti Putra Malaysia, 43400 UPM Serdang, Selangor, Malaysia; ^4^Department of Crop Science, Faculty of Agriculture, Universiti Putra Malaysia, 43400 UPM Serdang, Selangor, Malaysia

## Abstract

Molecular characterization of oil palm germplasm is crucial in utilizing and conserving germplasm with promising traits. This study was conducted to evaluate the genetic diversity structures and relationships among 26 families of MPOB-Senegal oil palm germplasm using thirty-five microsatellite markers. High level of polymorphism (*P* = 96.26%), number of effective allele (*N*_e_ = 2.653), observed heterozygosity (*H*_o_ = 0.584), expected heterozygosity (*H*_e_ = 0.550), total heterozygosity (*H*_T_ = 0.666), and rare alleles (54) were observed which indicates that MPOB-Senegal germplasm has a broad genetic variation. Among the SSR markers, sMo00053 and sMg00133 were the most informative markers for discrimination among the MPOB-Senegal oil palm germplasm for having the highest private alleles and the rare alleles. For selection and conservation, oil palm populations with high rare alleles and Nei's gene diversity index should be considered as these populations may possess unique genes for further exploitation.

## 1. Introduction

The African oil palm (Elaeis guineensis) is the most productive among oil-bearing palms. It is endemic largely in West and Central Africa's tropical lowlands, occurring extensively from 16°N in Senegal to 15°S in Angola [1]. Malaysia is the second-largest producer of oil palm after Indonesia, with a yearly production of 21 million metric tons. The current planting materials in Malaysia were derived from Deli and AVROS crosses to produce a Deli population derived from four palms introduced to Bogor in 1848 [1]. Oil palm breeders recognize the narrowness of the genetic base, and it has been established that the extreme narrowness of gene pool is the main obstacle in oil palm breeding program [2]. The Malaysian Palm Oil Board (MPOB) has extensively collected wild oil palm germplasm from 11 African countries for maintenance at the field gene bank at MPOB Research Station in Kluang to broaden the genetic bases of the current planting materials. Evaluation of genetic diversity based on morphology and physiology characteristics has been carried out on these germplasms. However, insufficient information due to low polymorphism, long juvenile phase, and genotype by environment interaction has hindered effective characterization of the germplasm. Hence, molecular markers are unique in assessing genetic diversity due to less influence of environmental factors [3].

Over time, the oil palm germplasm collections were screened using various molecular markers to evaluate and investigate the genetic variability between and within the population. Hayati et al. [4] studied the genetic diversity of MPOB germplasm collections using isoenzyme analysis. Molecular markers such as Amplified Fragment Length (AFLP) markers [5], Restriction Fragment Length Polymorphism (RFLP) [6], and simple sequence repeat (SSR) markers [7–9] have been extensively used to investigate genetic variation within MPOB germplasm collections. However, despite the molecular assessments, these studies have yet to cover all the germplasm populations. The evaluations should be extended to estimate each germplasm population genetic variation and relation [9]. Currently, simple sequence repeat (SSR) markers are one of the most promising molecular markers for understanding the genetic diversity and structure of oil palm with high specificity and polymorphism [10, 11], codominance [12], and relative abundance throughout the genome [13, 14].

Molecular characterization of MPOB-Senegal oil palm germplasm is yet to be evaluated among and within the populations. Also, MPOB-Senegal germplasm populations are adapted to dry weather conditions from their original collection site, which implies that they may possess drought-tolerant characteristics. Therefore, for further exploitation and understanding of valuable genes, genetic diversity and population structures of Senegal germplasm collections should be evaluated using the simple sequence repeat (SSR) marker. Hence, this study was conducted to evaluate the genetic diversity and population structures of MPOB-Senegal oil palm germplasm using microsatellite markers.

## 2. Materials and Methods

### 2.1. Planting Materials

Germplasm collection was carried out between July and August 1993 with assistance from the Ministry of Agriculture, Senegal. A total of 104 bunch samples belonging to eight different populations distributed across the southern and northern parts of Senegal were collected (Table 1) and planted in June 1996 at MPOB Research Station situated at Kluang in Johor, Malaysia. The planted bunch samples were categorized as trial 0.352.

### 2.2. DNA (Deoxyribonucleic Acid) Extraction

The molecular characterization was carried out on a total of twenty-six families from eight populations. Spear leaf (unopened leaves) samples were collected from each palm during the trial; the number of collected samples varied from 5 to 10 palms per family based on the availability of palms with a total sum of 222 palms obtained (Table 1). The spear leaf sample was shredded into small pieces and packed in a plastic bag, labeled and immersed in liquid nitrogen, and subsequently transferred into the freezer (-80°C). Four-day DNA extraction was carried out using the modified CTAB (cetyl trimethyl ammonium bromide) method. The concentration and purity of DNA were determined by measuring the absorbance at*λ* = 260.0 nm, 280.0 nm, and 350.0 nm using a spectrophotometer (Thermo Scientific, BoiMate 3S). All the samples were later diluted to 50 ng/*μ*l and stored at 20°C for subsequent PCR amplification use.

### 2.3. Genotyping by Multiplex PCR for Amplification of Microsatellite Markers

**Figure 1:**
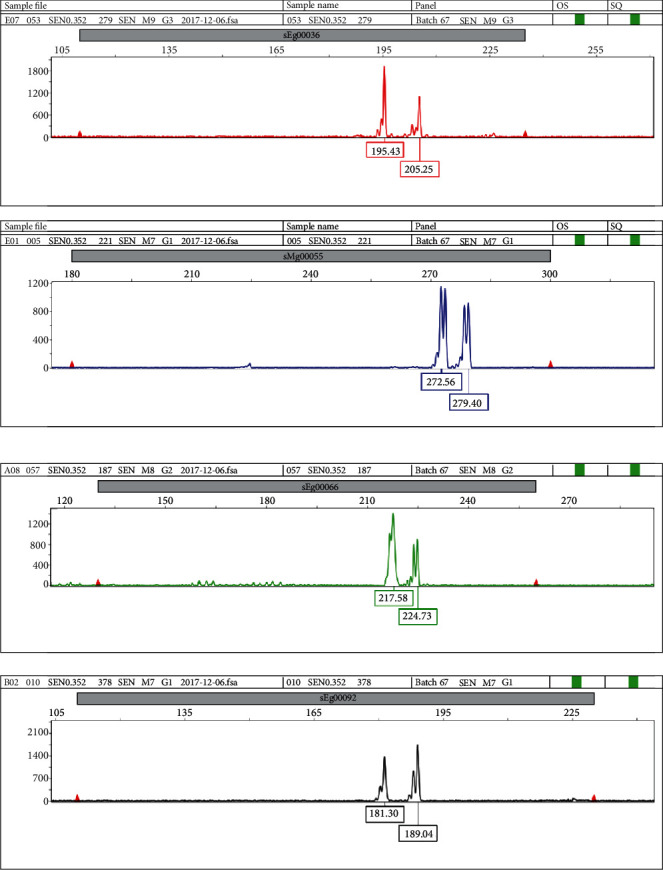
Electropherogram profiles from the ABI 3730 DNA Genetic Analyzer showing allele peaks and sizes of some SSR loci (sEg00036, sMg00055, sEg00066, sEg00092).

A total of 35 highly polymorphic and reproducible markers were selected for this study. The information of the 35 markers was presented in Supplementary Table 1. Out of the 35 markers used, 30 markers were developed at the Genomics Unit of ABBC-MPOB while the other 5 markers were developed at the French Center de Coopération Internationale en Recherche Agronomique pour le Dévelopement (CIRAD). Multiplex PCR protocol was conducted for genotyping the 26 families of MPOB-Senegal oil palm germplasm due to the large palm number (222) representing the families. For multiplex PCR reaction, a combination of four primers was designed. Every forward primer was M13-tailed and labeled with one of four florescent M13-dyes, viz., NED, FAM, VIC, and PET, to identify the multiplexing of the four markers for scoring of band pattern. Different dye colours distinguished the markers and related alleles in the output data where the band sizes overlapped (Figure 1). The total reaction of each polymerase reaction was 10 *μ*l, comprising 50 ng of genomic DNA, 6.625 *μ*l of MilliQ water, 1× PCR standard buffer (NEB, USA), 0.2 *μ*l of 10 mM deoxynucleotide triphosphates (dNTPs) (NEB, USA), 0.025 *μ*l of each of the M13-tailed forward primers and untailed reverse primers for every primer pair, 0.025 *μ*l dye, and 0.1 *μ*l of Taq DNA polymerase (5 U/*μ*l) (NEB, USA). PCR was performed in a Perkin Elmer 9600 Thermocycler following an initial denaturation temperature at 95°C for 3 minutes, followed by 35 cycles at 95°C for 30 seconds, primer annealing for 30 sec, and an extension temperature of 72°C for 30 minutes, terminated by a final extension at 72°C for 2 min. The amplified PCR products were resolved using 1% agarose gel and run in a horizontal electrophoresis system to check the band sizes using a 100 bp ladder. The DNA fragment on agarose gel was documented using Gel Imager® (GelDocTM XR, Bio-Rad Lab. Inc., Hercules, CA, USA). DNA concentration was measured by using a NanoDrop spectrophotometry machine (ND1000 Spectrophotometer USA). Four PCR products for different primers labeled with different fluorescent dyes were pooled and multiplexed with the new PCR plate. The pooled PCR products (2 *μ*l) were combined with 7.84 *μ*l of formamide (Applied Biosystems, Foster City, CA) and 0.16 *μ*l of the Gene Scan 500 (-35, -250, -340) LIZ size standard (Applied Biosystems, Foster City, CA). The samples were inserted in a 96-well PCR microplate and heated for 3 minutes. The sample plate was kept at 4°C before automated capillary electrophoresis using an ABI 3730 DNA Genetic Analyzer.

### 2.4. Statistical Analysis

SSR data were analyzed using Genemapper 4.1 software to identify the allele sizes of each marker. Electropherogram profiles (sample plots) were generated, and allele sizes for the markers were exported as data table for genotyping. Genetic diversity parameters such as allelic frequency, number of alleles per locus (*N*_a_), effective allele number (*N*_e_), observed heterozygosity (*H*_o_), expected heterozygosity (*H*_e_), fixation indices (*F*_is_), percentage of polymorphism (%*P*), and number of private alleles were calculated using GenAlex 6.5 software [15]. POPGENE software was used for cluster analysis using the Neighbour-Joining (NJ) method to evaluate the genetic relationships among 26 families.

## 3. Results

### 3.1. Allelic Diversity

A total of 384 alleles were regenerated, the number of alleles per locus ranging from 4 to 21, with an average value of 10.971. Markers sMo00053 and sMg00133 detected the highest number of alleles (21), indicating that these markers are the most informative marker while sMg00027, sEg00189, and sEg00092 identified the least number of alleles, with each marker identifying 4, 5, and 6 alleles, respectively (Table 2). The number of different alleles (*N*_a_) detected across 26 families ranged from 2.038 to 6.231, with an average of 3.869 different alleles. SMo00131 detected the highest number of different alleles, whereas marker sEg00189 recorded the least number of differences at 2.038 (Table 2). Among the families, SEN07.03 and SEN06.08 exhibited the highest number of different alleles at 4.686 and 4.429, respectively, while SEN02.04 had the least number of different alleles at 3.029 (Table 3). On the other hand, significant variation was observed between the number of effective alleles (*N*_e_), estimated as the inverse of homozygosity, and the observed alleles. The number of effective alleles varied from 1.393 to 4.662, and sMo00131 was the most informative marker among the markers evaluated. Family SEN05.08 had the highest number of effective alleles (3.06) as shown in Table 3.

Diversity indices provide important information regarding the rarity and commonness of species in a community. Shannon's information index (*I*) ranged from 0.401 (sEg00189) to 1.597 (sMo00131) with an average value of 1.024 (Table 2), and the highest Shannon information index (*I*) was recorded in family SEN05.05 (1.181) (Table 3). High heterozygosity was observed among the MPOB-Senegal oil palm germplasm with an average observed heterozygosity (*H*_o_) of 0.584 (Table 2). Among the employed markers, the highest observed heterozygosity (*H*_o_) was recorded in locus sEg00009. The expected heterozygosity (*H*_e_) among the 35 loci for 26 families varied from 0.236 in sEg00189 to 0.755 in sMo00131 while the average expected heterozygosity was 0.550.

Among the families, the lowest *H*_o_ was observed in SEN12.03 while the highest was recorded in SEN06.08. The highest *H*_e_ was recorded in SEN05.05, and the lowest was observed in SEN02.04. The observed heterozygosity was high, indicating the occurrence of a wide genetic diversity among the families. The chi-square test was employed in the determination of the differences between the observed (*H*_o_) and the expected (*H*_e_) heterozygosity of 26 families of MPOB-Senegal oil palm germplasm (Table 3). There was no significant difference between the observed heterozygosity and Hardy-Weinberg heterozygosity except for the SEN06.08 family. This shows no significant difference in the genetic variation among the families of MPOB-Senegal oil palm germplasm except SEN06.08 which may not be fully representative of all the alleles of the family. The average of total heterozygosity (*H*_T_) for the 26 families was 0.666, suggesting a high value of genetic diversity among the families. The highest total genetic diversity (*H*_T_) was observed in primers sMo00131 and mEgCIR0369 with values of 0.859 and 0.855, respectively (Table 2). The percentage of polymorphism among the families varied from 85.71 to 100% with an average of 96.26%, indicating a high level of polymorphism and wide genetic variations among the families.

The entire 35 amplified SSR markers were polymorphic with the highest level (100%) across the families observed in SEN 02.09, SEN03.07, SEN05.04, SEN05.05, SEN05.08, SEN06.08, SEN07.03, SEN10.05, and SEN12.03. The lowest polymorphism level was recorded in SEN02.04 and SEN10.03 with 85.71% and 88.57% polymorphism. These results showed that most families from the southern part have higher genetic diversity than the northern part of Senegal (Table 3).

### 3.2. Fixation Indices and Estimates of *N*_m_ over 26 Families of MPOB-Senegal Oil Palm Germplasm for 35 Loci

The inbreeding coefficient within the families (*F*_is_) per locus varied from -0.438 (sEg00061) to 0.510 (sMg00234) with a negative mean of – 0.050. These negative *F*_is_ indicate an excess of heterozygosity as observed in 23 loci. However, positive *F*_is_ values were observed in 12 loci which indicate less heterozygosity. Furthermore, the *F*_it_ (the inbreeding coefficient at total families) values ranged from -0.325 (sEg00061) to 0.618 (sMg00234) with an average value of 0.127. Positive *F*_it_ values were recorded in most of the loci except for 6 loci (sMg00055, sEg00009, sEg00061, sMo00136, sMo131, and sMg00025). The genetic differentiation (*F*_st_) varied from 0.091 (sEg00092) to 0.363 (sEg00126) with a mean value of 0.174. This result indicates that the genetic material can be shared among the families (Table 4). The estimation of *N*_m_ ranged from 0.438 (sEg00126) to 2.916 (sEg0006) with a mean value of 1.338, and this can be considered as high according to Bakoumé et al. [16] who described *N*_m_ value > 1 as high. *N*_m_ decreases with increasing *F*_st_ because greater differentiation between populations corresponds to lower levels of gene flow, thus, indicating that *N*_m_ is inversely related to *F*_st_. Marker sEg00126 detected the lowest *N*_m_, while also recording the highest *F*_st_. On the other hand, sEg0066 detected the highest *N*_m_ while low *F*_st_ was recorded by the marker (Table 4).

### 3.3. Private and Rare Allele

In this study, a total of 83 private alleles occurred in most of the families of MPOB-Senegal oil palm germplasm except in SEN05.08 and SEN07.04. The distributions of rare alleles per locus among the families and the frequencies are presented in Supplementary Table 2. The private and rare alleles varied in all the polymorphic markers in different families. Moges et al. [17] reported that private alleles or rare alleles unique in geographic regions are useful in comparing genetic variation between the species and populations. The number of private alleles detected by markers varied from 1 to 7 among the families as presented in Supplementary Table 2. Among the families, SEN05.03, SEN10.03, and SEN12.03 had the highest number of private alleles. Among the polymorphic markers, sMo00053 and sMg00133 were the most informative markers, detecting the highest private number of alleles.

The frequency of private allele varied from 0.050 to 0.667 at each locus of the 35 markers of 26 families (Supplementary Table 3). According to Brown (1978), the alleles can be categorized as “rare” if the frequency is more than 0.100 (10%) and presented in bold font to be identified from private alleles. Among the employed 35 loci, 32 loci could detect the private alleles, whereas three loci sEg00041, sEg00189, and sMg00027 could not detect the private alleles. Based on the allele frequency results, 53 rare alleles were detected by 25 loci from 21 families. Based on the results, SEN07.05 and SEN12.03 have the highest rare alleles among the families. The most informative markers were sMo00053 and sMg00133, scoring the highest rare allele (5). This observation was in agreement with the study reported by Zulkifli et al. [9] and Bakoumé et al. [16], who recorded rare alleles in some Senegal germplasm populations. Upadhyaya et al. [18] reported that unique alleles occur solely in one accession or one group of accessions and are not found in any other. Unique alleles can be used to discriminate the families among themselves. Of 83 private alleles, three unique alleles detected by locus (found in one family but not at any other families), viz., 14/mEgCIR0369, 16/sMo00131, and 18/sMo00053, were observed in SEN12.03, SEN04.03, and SEN12.01, respectively.

### 3.4. Analysis of Molecular Variance (AMOVA) of 26 Families of MPPOB-Senegal Oil Palm Germplasm

The AMOVA results indicated that the highest genetic variation was 83% and occurred within the families, while only 17% genetic variation was observed among the families at *P* < 0.001 level. The variance component varied from 5.357 to 25.484 (Table 5). Based on the analysis results, a rich genetic diversity was observed in individuals within the families, indicating that their genetic variation was larger than among the families.

### 3.5. Genetic Relatedness among the Families of MPOB-Senegal Oil Palm Germplasm

The genetic distance among the 26 families based on Nei's genetic distance is summarized in Table 6. The genetic distance among the 26 families varied from 0.100 to 0.557, while the average genetic distance was 0.315. The minimum genetic distance (0.100) was observed between SEN02.04 and 02.06, whereas the maximum genetic distance (0.557) was recorded between SEN05.03 and SEN12.01. The genetic distance between the families from the same population was low compared to families from different populations. The minimum average genetic distance was observed among the families of population ten, whereas the maximum average was recorded among the families of population 3. The families' genetic distance results revealed that the widest genetic distance occurred between populations 5 and 12.

### 3.6. Cluster Analysis

**Figure 2:**
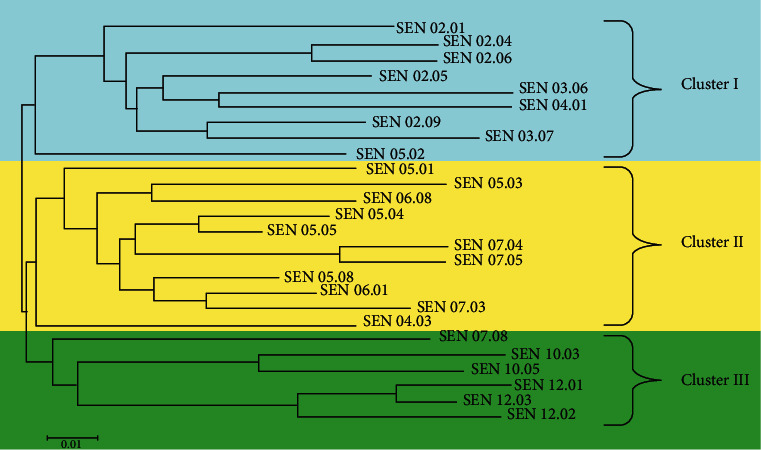
Neighbour-Joining (NJ) tree constructed for SSR data based on 26 families of MPOB-Senegal germplasm.

Datasets of 35 SSR markers used in the study of 26 families of MPOB-Senegal germplasm were analyzed using Pop tree software, and the dendrogram was constructed for the result obtained (Figure 2). The dendrogram grouped the families into three major clusters. Cluster I consists of two subclusters, and the first subcluster comprises eight families (SEN02.01, SEN02.04, SEN02.06, SEN02.05, SEN03.06, SEN04.01, SEN02.09, and SEN03.07) while the second subcluster is a single cluster which was shown as SEN05.02. Cluster II is the largest cluster comprising 11 out 26 families, forming two subclusters. The first subcluster consists of 10 members, namely, SEN05.01, SEN05.03, SEN06.08, SEN05.04, SEN05.05, SEN07.04, SEN07.05, SEN05.08, SEN06.01, and SEN07.03 while the second subcluster stands individually, consisting solely of SEN04.03. A total of 6 families formed cluster III, and these were divided into two subclusters. The first subcluster stands as a singleton with SEN07.08 as the only family while the second subcluster consists of four families, viz., SEN10.03, SEN10.05, SEN12.01, SEN12.02, and SEN12.03. Families of population 12, situated at the northern part of Senegal, were grouped with families from population 10, and both formed cluster III. Moreover, SEN07.08 was also clustered into cluster III due to the genetic similarity among the cluster members. In general, the grouping pattern of families was not consistent with the geographical distributions of sample collections.

## 4. Discussion

### 4.1. Allelic Diversity

The evaluation of genetic diversity and relatedness among 26 families of MPOB-Senegal oil palm germplasm using 35 polymorphic SSR markers showed 384 alleles with a mean value of 10.97 alleles per locus. The number of alleles per marker ranged from 4 to 21 alleles. This was higher than the total number of alleles reported by Shah et al. [19] who detected 6 alleles in a genetic diversity assessment of germplasms from Africa. The total number of alleles observed in this study was also higher than that reported by Hayati et al. [4] and Maizura et al. [6], who reported 21 and 58 alleles, respectively, while evaluating the genetic diversity of 11 African oil palm germplasm using isozyme and RFLP, respectively. Similarly, the alleles reported in this study were higher than those reported in Singh et al. [13] and Ting et al. [20] who recorded a total number of 48 and 101 alleles using 10 and 15 EST-SSR markers, respectively, in the genetic diversity evaluation of African oil palm germplasm. The result was also higher than that reported by Zulkifli et al. [9] and Bakoumé et al. [8] in their respective evaluation of 11 African oil palm germplasms where 64 alleles and 209 alleles were reported using 10 and 16 SSR markers, respectively. Additionally, the alleles observed in this study result was higher than those observed by Arias et al. [21] where a total of 223 alleles were reported in Cameroon germplasm using 31 SSR, as well as the result of Arias et al. [22] where 195 alleles were reported in Angola germplasm when 30 SSR markers were used. This study indicated that SSR markers are more efficient than isozyme and RFLP markers in the evaluation of genetic diversity. Moreover, the accuracy of result obtained in a genetic diversity study is influenced by the number of markers utilized and sample size evaluated.

The mean percentages of polymorphic loci of MPOB-Senegal germplasm using 35 SSR markers were 96.26%. This was higher than the mean values reported by Hayati et al. [4] and Maizura et al. [6] where isozyme and RFLP were used in their respective studies. Hayati et al. [4] reported that the percentage of polymorphic loci for population 2 and 12 of Senegal germplasm was 71.4% and 57.1%, respectively, while Maizura et al. [6] observed 55.2% polymorphic loci for Senegal germplasm in the genetic diversity assessment of MPOB-African oil palm germplasm. High percentage of polymorphic loci indicates that SSR markers were effective for genetic diversity assessment compared to isozyme and RFLP, also confirming that the crop genetic diversity influenced molecular marker techniques.

The comparisons of results of the percentage of polymorphic loci have also been recorded among studies where microsatellites were used. A higher result was reported in the current study than Singh et al. [13], and Ting et al. [20] observed 90% polymorphism in Senegal oil palm germplasm. However, the present study's result was lower than that reported by Zulkifli et al. [9] and Arias et al. [23] who reported 100% polymorphic loci in the assessment of Senegal and Cameroon germplasm, respectively. A high number of alleles and percentage of polymorphism in the Senegal germplasm indicate that the germplasm has high genetic variability and the potential to be exploited in oil palm breeding program. Population 2 of Senegal germplasm was recommended for utilization in the oil palm improvement program due to its high average number of alleles per locus (*N*_a_ = 1.71), effective allele (*N*_e_ = 1.39), number of observed alleles (*H*_o_ = 0.224), and number of expected alleles (*H*_e_ = 0.232). In addition, population 12 also showed high *N*_a_ 1.57, *N*_e_ 1.28, *H*_o_ 0.171, and *H*_e_ 0.171. Moreover, Maizura et al. [6] reported that the number of alleles per locus (*N*_a_) was 1.7 and expected heterozygosity (*H*_e_) was 0.214 in the genetic diversity study of 11 African oil palm germplasms using RFLP. In the current study, these genetic variability results increased to the number of alleles per locus (*N*_a_ = 3.869), expected heterozygosity (*H*_e_ = 0.550). These genetic diversity measures increased with an increase in the number of samples covering all Senegal oil palm populations. The molecular technique used also influences the result of a genetic diversity study. Thus, these results agree with Ting et al. [20], who claimed that *N*_a_ and *N*_e_'s results were affected by the number of markers and sample size assay.

The observed number of alleles per locus (*N*_a_ = 3.869), effective alleles per locus (*N*_e_ = 2.653), observed heterozygosity (*H*_o_ = 0.584), and expected heterozygosity (0.550) were higher in this study compared to the previous study by Singh et al. [13] with the values of 2.6, 1.85, 0.42, and 0.53, respectively, which were observed among seven African oil palm germplasms using 10 EST-SSR. Similarly, the result was higher than that reported by Ting et al. [20] who used 15 EST-SSR markers in their study and reported heterozygosity (*H*_o_) of 0.434 and expected heterozygosity (*H*_e_) of 0.436 in Senegal germplasm. Meanwhile, the heterozygosity results of Madagascar, Gambia, Ghana, Congo, Cameroon, and Nigeria germplasm from the studies of Singh et al. [13] and Ting et al. [20] were lower than those of Senegal germplasm in the current study. Zulkifli et al. [9] evaluated the genetic diversity of MPOB germplasms using 10 EST-SSR and reported a mean value of 3.6 in the number of alleles per locus, 0.387 in observed heterozygosity (*H*_o_), and 0.496 in expected heterozygosity (*H*_e_) of Senegal germplasm. This was lower than the values recorded in MPOB-Senegal germplasm in the current study, where the mean number of alleles per locus (*N*_a_) = 3.869, the effective alleles per locus (*N*_e_) = 2.653, the observed heterozygosity (*H*_o_) = 0.584, and expected heterozygosity (*H*_e_) = 0.550. This result was equivalent to the results obtained in Cameroon, Congo, and Ghana where the mean value of *N*_a_ was 3.8 and was comparable with Madagascar (*N*_a_ = 3.7), Angola (*N*_a_ = 3.5), and Gambia (*N*_a_ = 2.7). Likewise, the mean heterozygosity of the current study on MPOB-Senegal oil palm germplasm (*H*_o_ = 0.584 and *H*_e_ = 0.550) was greater than the values (*H*_o_ = 0.387 and *H*_e_ = 0.495) recorded in Senegal germplasm by Zulkifli et al. [9]. However, in Bakoumé et al.'s [8] study on the genetic diversity of 10 African germplasms using 16 SSR, the number of observed and effective alleles per locus for population 5 of Senegal germplasm was 5.2 and 3, respectively. These results were higher than those recorded in the current study (*N*_a_ = 4.014, *N*_e_ = 2.821). However, the observed heterozygosity (*H*_o_ = 0.542) was lower compared to the current study. In this research, the observed heterozygosity (*H*_o_) values for all the 35 evaluated markers were higher than the expected heterozygosity (*H*_e_). This result corroborates the result of Bodia et al. [24] who used 18 SSR markers to evaluate the genetic diversity of date palm cultivars from Figuig oasis (Morocco) from which the *H*_o_'s value was higher than *H*_e_.

Senegal is located in the extreme part of West Africa [8]. The heterozygosity decreased from the countries such as Nigeria, Congo, Ghana, and Cameroon in the central region of West Africa to the marginal regions as Senegal, Gambia, and Madagascar [18]. Also, Maxted et al. [25] described that the genetic diversity in natural populations of plants decreases according to the geographical distance from their place of origin and these reports were consistent with the current results on Senegal germplasm which showed lower heterozygosity compared to those reported (*H*_o_ = 0.587, *H*_e_ = 0.649) by Arias et al. [21] in Cameroon germplasm. The value of Shannon's information index (*I*) recorded in the current study was higher (1.024) than that reported by Zhou et al. [26], who observed the average *I* of 0.6565 using a microsatellite marker to assess genetic diversity among the oil palm materials collected from Malaysia and China. The result was also comparable with Bharath et al. [27] who recorded a mean value of 1.35 in Shannon's information index using RAPD markers to investigate the genetic diversity among 60 areca nut palm germplasms collected from India and seven other Southeast Asian countries. Also, the mean value of Shannon's information index in this study was higher than the results recorded by Shah et al. [19] in five populations of Zaire (0.43, 0.46, 0.33, 0.35, and 0.21), one population of Cameroon (0.26), Tanzania (0.33), and Nigeria (034). Shannon's information index result using RAPD markers was lower than the results revealed by SSR markers. The presence of high-value Shannon's index further validates some diversity parameters such as *N*_e_, *H*_e_, and %*P* in understanding populations' genetic diversity. Among the 26 families, SEN05.05 showed the highest *I* value (1.181) as well as the highest *N*_e_ (3.036), *H*_e_ (0.621), and *P* (100%). On the other hand, SEN02.04 had the lowest *I* value and 12 families had Shannon's information index value lower than the trial mean. This Shannon's information index parameter could be used as an initial criterion to narrow down the number of accessions for evaluation [19] and could be combined with *N*_e_, *H*_e_, and %*P* information for use in establishing core germplasm.

### 4.2. Fixation Indices and Estimates of *N*_m_ over 26 Families of MPOB-Senegal Oil Palm Germplasm for 35 Loci

The fixation indices *F*_is_, *F*_it_, and *F*_st_ were estimated to measure the excess or deficit of heterozygosity in the entire families. The total genetic diversity (*F*_st_) among the 26 families was 0.174, and this was considered high according to Wright [28]. Negative *F*_is_ showed an excess of heterozygosity at 23 loci with a mean value of -0.050. The average gene flow (*N*_m_ = 1.338) in this study is considered high, as Slatkin reported [29]. These high gene flows could decrease genetic variation among the families because gene migration between distant populations can reduce the genetic differentiation among the populations [30, 31].

### 4.3. Private and Rare Allele

Moges et al. [29] reported that private alleles or rare alleles unique in geographic regions are useful in comparing genetic variation between the species and populations. In this study, the number of private alleles (83) and rare alleles (53) in MPOB-Senegal oil palm germplasm was relatively high. These results are similar to those reported by Hayati et al. [4] and Zulkifli et al. [9] who recorded rare alleles in Senegal germplasm using isozyme and SSR marker, respectively. The present study's result agrees with Bakoumé et al. [8], who observed rare alleles in almost all the localities of Senegal, which may indicate adaptive traits to low rainfall and dry weather condition. Rajora et al. [32] also described that the presence of rare alleles may refer to plant adaption to abiotic and biotic stress due to environmental conditions. Following this, a prevalence in numbers of rare alleles (53) in the current study may suggest that this germplasm possesses the traits related to low rainfall and dry weather.

According to Zeng et al. [33] and Upadhyaya et al. [18], three alleles in the current study were defined as unique alleles due to their occurrence solely in one family. The loci mEgCIR0369, sMo00131, and sMo00053 amplified the three unique alleles, namely, 14, 16, and 18, in SEN12.03, SEN04.03, and SEN12.01, respectively. From this, it can be said that mEgCIR0369, sMo00131, and sMo00053 have a higher capacity to distinguish the families which possess these unique alleles. This result corroborates the results reported by Swaray et al. [34], Sidhoum et al. [35], and Boukhari et al. [36] who observed highly informative and effectively differentiation among studies genotypes. Zeng et al. [33] assumed that unique alleles (or alleles not detected in commercial cultivars) in breeding lines and landraces of rice have important function in their adaptation to saline soils. Therefore, this study's unique alleles are important in investigating specific genes in the genome area for oil palm adaption to low rainfall and dry weather conditions.

### 4.4. Analysis of Molecular Variance (AMOVA) of 26 Families of MPPOB-Senegal Oil Palm Germplasm

The analysis of molecular variance (AMOVA) shows that genetic variations were greater within the families. The total genetic diversity (*F*_st_) among the 26 families was 0.174 and can be considered as high according to Wright [28]. Also, an excess of heterozygosity was revealed by a negative value of *F*_is_. Based on segregating genetic diversity, a considerable genetic diversity within the families would be applied in the oil palm improvement program.

### 4.5. Genetic Relatedness among the Families of MPOB-Senegal Oil Palm Germplasm

The genetic correlation among the families is quite variable, ranging from 0.100 to 0.557. It is not surprising that the lowest genetic distance value (0.100) was recorded between SEN02.04 and SEN02.06; this is because the two families belong to the same population, namely, population 2. Generally, there is a strong relationship between genetic distance and geographical locations [9, 13]. The longest genetic distance between SEN05.03 and SEN12.01 could be due to the geographical distance, where SEN05.03 originates in the southern part whereas SEN12.01 belongs to the northern part. Families separated by greater distance were more genetically divergent than those families that are geographically adjacent, indicating the occurrence of a stronger internal genetic variation. The evidence of genetic distance among the families is important for breeders in the exploitation of heterosis effect in oil palm breeding program.

### 4.6. Cluster Analysis

The UPGMA dendrogram was constructed that showed the genetic similarity and dissimilarity among the families. Among the total 8 populations, populations 2, 3, 4, 5, 6, 7, and 10 were situated in the southern part of Senegal whereas population 12 was located in the northern part, separated by Gambia. The populations in the southern part were within an 80 km radius. Populations 2 and 3 were collected around 45 km from Ziguinchor while population 5 was collected around 17 km from Ziguinchor whereas population 4 was from Dar Salam. Among the three main clusters as revealed by the dendrogram, families from the same population were not assembled in the same cluster; however, a grouping of families from different populations was formed. The families of populations 2 and 3 were grouped in cluster I. Additionally, families SEN04.01 and SEN05.02 were contained in cluster I while the rest of the families from populations 4 and 5 were grouped with families of populations 6 and 7 in cluster II. This is due to the limited geographical distance in the collection sites of populations 2, 3, 4, 5, 6, 7, and 10 (not more than 80 km).

On the other hand, two families of population 10 and family SEN07.08 were grouped with three northern outlying families from population 12 to form cluster III, which may be due to the genetic similarity among the families and the high number of migrants (*N*_m_ = 1.338). This result is similar to that reported by Arias et al. [21] who described that genetic similarity or homogeneity of genetic variation could be increased due to high number of migrants (*N*_m_). These results indicated a weak genetic differentiation among the families, and this is consistent with the AMOVA result which shows lesser genetic variation among the families than among the individuals. Based on the results of the cluster, distributions of different families are a mixed type. This is in agreement with Oladosu et al. [37] and Oladosu et al. [38] who reported that genetic diversity is related to geographical diversity but not necessarily and directly associated with geographical distribution. The results are also consistent with the results of Arias et al. [21] and Arias et al. [22] in their studies on the collections of Cameroon and Sierra Leone oil palm populations, where it was stated that there was no distinct relation between geographical locations and phenotypic variation. However, the result is in contrast with the results of Kularatne et al. [5], Zulkifli et al. [9], and Bakoumé et al. [8] who reported a strong relationship between genetic distance and geographical location of African oil palm germplasms using AFLP, isozymes, RFLP, and SSR in their respective studies.

## 5. Conclusions

The simple sequence repeat markers are useful in detecting the genetic variability and relationship among MPOB-Senegal oil palm germplasm. It shows a high degree of sensitivity for discriminating genetic variability, thereby providing ample opportunity for further genetic improvement. This will assist oil palm breeders in identifying redundancies in the collection and development strategies for field conservation. The presence of relatively high different allele, effective alleles, and heterozygosity indicates that MPOB-Senegal germplasm possesses unique traits which could be used in the future breeding program. Among the SSR markers employed, sMo00053 and sMg00133 were the most informative markers for MPOB-Senegal oil palm germplasm due to their capability to detect both the highest private alleles and the rare alleles. SEN07.05 and SEN12.03 were unique families due to the highest occurrence of rare alleles. Also, SEN05.03 and SEN12.03 families had the highest private alleles. The information obtained from this study could be vital in core collection establishment without any loss of genetic variations.

## Figures and Tables

**Table 1:** Geographical origins and palm number represent in each family of MPOB-Senegal oil palm germplasm.

No.	Family name	Location	No. of palms	Palm number
1	SEN02.01	45 km from Ziguinchor	7	172, 213, 229, 233, 267, 287, 292
2	SEN02.04		6	67, 74, 119, 161, 165, 282
3	SEN02.05		6	20, 39, 68, 83, 86, 231
4	SEN02.06		10	1, 9, 88, 138, 141, 243, 364,383, 386, 410
5	SEN02.09		10	73, 105, 118, 124, 289, 343, 365, 368, 379, 408
6	SEN03.06		7	17, 192, 225, 250, 253, 277, 301
7	SEN03.07		10	29, 35, 37, 72, 254, 286, 353, 357, 359, 366
8	SEN04.01	Dar Salam	10	84, 94, 112, 169, 196, 200, 216, 248, 338, 369
9	SEN04.03		10	22, 55, 77, 79, 109, 184, 206, 215, 294, 348
10	SEN05.01	Babadinka 17 km de Ziguinchor	10	120, 132, 227, 251, 262, 271, 335, 342, 351, 363
11	SEN05.02		5	21, 69, 145, 249, 274
12	SEN05.03		10	128, 135, 239, 263, 297, 314, 333, 358, 367, 373
13	SEN05.04		6	48, 80, 140, 143, 244, 272
14	SEN05.05		9	28, 66, 78, 102, 133, 164, 171, 174, 264,
15	SEN05.08		10	32, 93, 167, 176, 241, 247, 319, 344, 370, 396
16	SEN06.01	Diarone	7	96, 149, 252, 258, 276, 302, 305
17	SEN06.08		10	45, 91, 163, 187, 207, 214, 228, 283, 336, 382
18	SEN07.03	Djiticoumbon	10	10, 89, 114, 130, 194, 376, 392, 398, 400, 402
19	SEN07.04		9	31, 47, 58, 85, 115, 125, 246, 261, 298
20	SEN07.05		7	98, 117, 139, 146, 157, 210, 257
21	SEN07.08		7	56, 104, 127, 166, 236, 245, 255
22	SEN10.03	Ebaniko	10	23, 87, 110, 134, 173, 189, 278, 310, 323, 407
23	SEN10.05		10	24, 75, 131, 177, 186, 208, 212, 230, 280, 334
24	SEN12.01	Mboro	9	2, 12, 26, 70, 147, 170, 193, 242, 273
25	SEN12.02		7	4, 30, 54, 95, 183, 201, 222
26	SEN12.03		10	3,113, 126, 182, 203, 279, 340, 361, 389, 405
Total			222	

**Table 2:** Allelic diversity indices of evaluated markers in Senegal oil palm germplasm.

Marker	*N*	*N*_a_	*N*_e_	*I*	*H*_o_	*H*_e_	*H*_T_
sMg00093	13	3.346	2.147	0.871	0.542	0.485	0.612
sEg00035	7	2.154	1.393	0.417	0.155	0.242	0.281
sEg00151	8	3.192	2.007	0.752	0.465	0.407	0.536
sMg00050	13	3.885	2.614	1.034	0.492	0.555	0.783
sMo00023	10	3.962	2.783	1.113	0.659	0.607	0.784
sEg00126	11	3.115	1.859	0.731	0.277	0.409	0.642
sEg00041	9	4.885	3.311	1.322	0.677	0.675	0.819
sEg00025	7	2.654	2.177	0.808	0.594	0.498	0.613
sMg00055	9	2.923	1.964	0.776	0.634	0.457	0.536
sEg00066	8	3.269	2.141	0.878	0.549	0.511	0.663
sEg00036	10	4.423	2.759	1.153	0.632	0.601	0.715
sEg00092	6	2.577	1.415	0.476	0.283	0.257	0.283
sEg00189	5	2.038	1.423	0.401	0.234	0.236	0.289
sMg00234	11	3.654	2.748	1.072	0.296	0.604	0.775
sMo00077	9	2.769	1.659	0.561	0.247	0.303	0.370
sMg00192	13	4.885	3.207	1.308	0.644	0.666	0.825
sMg00077	10	3.846	2.494	1.037	0.549	0.565	0.736
sEg00009	10	4.577	3.496	1.331	0.909	0.702	0.792
sEg00061	7	3.385	2.673	1.063	0.892	0.620	0.673
sMo00081	10	4.115	2.880	1.144	0.584	0.610	0.727
mEgCIR0882	12	4.962	3.490	1.336	0.763	0.678	0.791
sMg00136	9	4.038	3.084	1.185	0.787	0.650	0.739
sMo00131	16	6.231	4.662	1.597	0.869	0.755	0.859
mEgCIR3546	13	4.077	2.843	1.121	0.717	0.609	0.727
sMg00025	9	3.192	2.498	0.978	0.791	0.577	0.649
sMo00053	21	4.192	2.820	1.085	0.524	0.567	0.771
mEgCIR3878	15	5.231	3.487	1.339	0.680	0.667	0.763
sMg00133	21	5.231	3.637	1.385	0.647	0.692	0.822
sMg00027	4	2.846	2.200	0.855	0.577	0.524	0.624
sMg00227	10	4.000	2.533	1.045	0.667	0.565	0.672
sMg00205	14	3.385	2.231	0.907	0.565	0.520	0.605
sMo00121	8	2.885	1.831	0.668	0.413	0.377	0.476
mEgCIR3282	18	5.154	3.496	1.372	0.631	0.690	0.797
mEgCIR0369	15	5.654	3.978	1.483	0.834	0.728	0.855
sMg00147	13	4.692	2.925	1.222	0.657	0.632	0.721
Mean	10.97	3.869	2.653	1.024	0.584	0.550	0.666

*N*: allele number per marker; *N*_a_: number of different alleles; *N*_e_: number of effective alleles; *I*: Shannon's information index; *H*_e_: Nei's gene diversity index; *H*_o_: number of observed alleles.

**Table 3:** Allelic diversity indices in families of Senegal oil palm germplasm.

Family	*N*_a_	*N*_e_	*I*	*H*_o_	*H*_e_	%*P*	Chi^2^ test *H*_o_ vs. *H*_e_
SEN02.01	3.486	2.425	0.923	0.558	0.503	94.29%	0.81
SEN02.04	3.029	2.251	0.832	0.567	0.474	85.71%	0.94
SEN02.05	3.371	2.540	0.950	0.542	0.523	91.43%	0.93
SEN02.06	4.057	2.673	1.036	0.552	0.545	94.29%	0.49
SEN02.09	4.343	2.772	1.079	0.591	0.560	100.00%	0.33
SEN03.06	3.943	2.956	1.100	0.536	0.583	97.14%	0.42
SEN03.07	4.257	2.681	1.078	0.563	0.568	100.00%	0.36
SEN04.01	3.571	2.385	0.912	0.484	0.494	94.29%	0.84
SEN04.03	3.914	2.598	1.018	0.619	0.551	97.14%	0.31
SEN05.01	4.400	2.873	1.143	0.620	0.598	97.14%	0.13
SEN05.02	3.686	2.886	1.084	0.697	0.594	97.14%	0.57
SEN05.03	4.286	2.971	1.149	0.631	0.609	97.14%	0.33
SEN05.04	3.429	2.565	0.995	0.600	0.558	100.00%	0.16
SEN05.05	4.314	3.036	1.181	0.610	0.621	100.00%	0.56
SEN05.08	3.971	2.592	0.992	0.614	0.530	100.00%	0.28
SEN06.01	3.886	2.825	1.068	0.626	0.571	97.14%	0.29
SEN06.08	4.429	2.864	1.147	0.709	0.600	100.00%	0.04
SEN07.03	4.686	2.947	1.162	0.670	0.593	100.00%	0.08
SEN07.04	4.029	2.852	1.035	0.583	0.538	94.29%	0.38
SEN07.05	3.686	2.587	0.982	0.583	0.531	91.43%	0.47
SEN07.08	3.571	2.610	1.003	0.584	0.554	94.29%	0.88
SEN10.03	3.571	2.380	0.918	0.490	0.500	88.57%	0.78
SEN10.05	4.171	2.818	1.102	0.586	0.586	100.00%	0.59
SEN12.01	3.743	2.395	0.947	0.525	0.510	97.14%	0.59
SEN12.02	3.200	2.301	0.893	0.564	0.508	94.29%	0.77
SEN12.03	3.571	2.203	0.887	0.476	0.490	100.00%	0.43
Mean	3.869	2.653	1.024	0.584	0.550	96.26%	

SEN: Senegal; *N*_a_: number of different alleles; *N*_e_: number of effective alleles; *I*: Shannon's information index; *H*_e_: Nei's gene diversity index; *H*_o_: number of observed alleles; %*P*: percentage of polymorphism.

**Table 4:** Fixation indices and estimates of *N*_m_ over all families of MPOB-Senegal oil palm germplasm for each locus.

Locus	*F*_is_	*F*_it_	*F*_st_	*N*_m_
sMg00093	-0.116	0.115	0.207	0.955
sEg00035	0.357	0.447	0.139	1.548
sEg00151	-0.142	0.133	0.241	0.788
sMg00050	0.114	0.372	0.291	0.610
sMo00023	-0.086	0.159	0.226	0.857
sEg00126	0.323	0.569	0.363	0.438
sEg00041	-0.003	0.173	0.176	1.174
sEg00025	-0.193	0.030	0.187	1.084
sMg00055	-0.389	-0.182	0.149	1.429
sEg00066	-0.075	0.171	0.229	0.839
sEg00036	-0.052	0.116	0.160	1.316
sEg00092	-0.100	0.001	0.091	2.483
sEg00189	0.012	0.192	0.182	1.121
sMg00234	0.510	0.618	0.221	0.879
sMo00077	0.184	0.332	0.181	1.132
sMg00192	0.034	0.220	0.192	1.049
sMg00077	0.027	0.253	0.232	0.825
sEg00009	-0.295	-0.147	0.114	1.949
sEg00061	-0.438	-0.325	0.079	2.916
sMo00081	0.043	0.197	0.161	1.302
mEgCIR0882	-0.126	0.036	0.144	1.489
sMg00136	-0.212	-0.065	0.121	1.818
sMo00131	-0.151	-0.011	0.122	1.805
mEgCIR3546	-0.177	0.013	0.161	1.298
sMg00025	-0.371	-0.220	0.110	2.022
sMo00053	0.076	0.321	0.265	0.693
mEgCIR3878	-0.020	0.108	0.126	1.738
sMg00133	0.066	0.213	0.157	1.340
sMg00027	-0.100	0.076	0.161	1.306
sMg00227	-0.181	0.007	0.159	1.324
sMg00205	-0.086	0.066	0.140	1.536
sMo00121	-0.096	0.131	0.207	0.957
mEgCIR3282	0.086	0.209	0.135	1.608
mEgCIR0369	-0.146	0.024	0.148	1.439
sMg00147	-0.040	0.088	0.123	1.775
	-0.050	0.127	0.174	1.338

*F*_is_ = (mean *H*_e_–mean *H*_o_)/mean *H*_e_; *F*_it_ = (*H*_T_–mean *H*_o_)/*H*_T_; *F*_st_ = (*H*_T_ − mean *H*_e_/*H*_T_; *N*_m_ = gene flow.

**Table 5:** Analysis of molecular variance.

Source of variation	df	SS	MS	Est. Var.	%	*P* value
Between families	25	1778.787	71.151	5.357	17%	<0.001
Within amilies	196	4994.821	25.484	25.484	83%	<0.001
Total	221	6773.608		30.841	100%	

df: degree of freedom; SS: sum square; MS: mean square; Est. Var.: estimated variance.

**Table 6:** Pairwise population matrix of Nei genetic distance among the 26 families of MPOB-Senegal oil palm germplasm.

	SEN02.01	SEN02.04	SEN02.05	SEN02.06	SEN02.09	SEN03.06	SEN03.07	SEN04.01	SEN04.03	SEN05.01	SEN05.02	SEN05.03	SEN05.04
SEN02.01	0.000												
SEN02.04	0.252	0.000											
SEN02.05	0.212	0.281	0.000										
SEN02.06	0.163	0.100	0.197	0.000									
SEN02.09	0.215	0.223	0.217	0.171	0.000								
SEN03.06	0.231	0.229	0.223	0.168	0.187	0.000							
SEN03.07	0.270	0.381	0.378	0.283	0.246	0.278	0.000						
SEN04.01	0.277	0.302	0.272	0.228	0.290	0.190	0.391	0.000					
SEN04.03	0.296	0.267	0.337	0.258	0.295	0.275	0.428	0.226	0.000				
SEN05.01	0.247	0.262	0.323	0.224	0.310	0.278	0.403	0.258	0.233	0.000			
SEN05.02	0.225	0.356	0.417	0.288	0.402	0.358	0.396	0.398	0.315	0.291	0.000		
SEN05.03	0.380	0.403	0.435	0.389	0.392	0.333	0.460	0.424	0.406	0.264	0.394	0.000	
SEN05.04	0.297	0.381	0.427	0.306	0.429	0.392	0.435	0.407	0.342	0.209	0.284	0.374	0.000
SEN05.05	0.298	0.323	0.334	0.266	0.363	0.339	0.417	0.349	0.303	0.233	0.344	0.352	0.208
SEN05.08	0.202	0.274	0.252	0.189	0.217	0.275	0.374	0.283	0.250	0.214	0.249	0.314	0.217
SEN06.01	0.278	0.303	0.327	0.243	0.288	0.317	0.381	0.337	0.305	0.258	0.269	0.339	0.292
SEN06.08	0.309	0.356	0.405	0.295	0.344	0.375	0.405	0.389	0.372	0.300	0.309	0.325	0.331
SEN07.03	0.242	0.341	0.296	0.271	0.289	0.346	0.375	0.370	0.391	0.279	0.286	0.355	0.266
SEN07.04	0.288	0.375	0.348	0.299	0.356	0.390	0.468	0.370	0.405	0.274	0.398	0.448	0.317
SEN07.05	0.270	0.322	0.399	0.238	0.341	0.311	0.428	0.317	0.352	0.255	0.331	0.391	0.304
SEN07.08	0.217	0.287	0.341	0.228	0.256	0.238	0.374	0.307	0.245	0.202	0.269	0.339	0.318
SEN10.03	0.325	0.392	0.366	0.290	0.381	0.364	0.414	0.386	0.311	0.311	0.338	0.513	0.459
SEN10.05	0.249	0.308	0.341	0.246	0.385	0.306	0.442	0.336	0.300	0.256	0.266	0.416	0.319
SEN12.01	0.361	0.406	0.447	0.285	0.425	0.454	0.532	0.409	0.386	0.270	0.420	0.557	0.378
SEN12.02	0.255	0.385	0.343	0.263	0.365	0.353	0.487	0.365	0.356	0.269	0.306	0.441	0.398
SEN12.03	0.317	0.339	0.369	0.235	0.342	0.337	0.464	0.383	0.347	0.294	0.349	0.509	0.390
Family	SEN05.05	SEN05.08	SEN06.01	SEN06.08	SEN07.03	SEN07.04	SEN07.05	SEN07.08	SEN10.03	SEN10.05	SEN12.01	SEN12.02	SEN12.03
SEN05.05	0.000												
SEN05.08	0.191	0.000											
SEN06.01	0.279	0.167	0.000										
SEN06.08	0.312	0.238	0.201	0.000									
SEN07.03	0.292	0.163	0.163	0.252	0.000								
SEN07.04	0.194	0.204	0.319	0.330	0.231	0.000							
SEN07.05	0.255	0.204	0.246	0.252	0.217	0.128	0.000						
SEN07.08	0.275	0.241	0.254	0.339	0.246	0.296	0.246	0.000					
SEN10.03	0.454	0.369	0.382	0.433	0.384	0.420	0.405	0.294	0.000				
SEN10.05	0.344	0.272	0.316	0.323	0.300	0.335	0.293	0.235	0.157	0.000			
SEN12.01	0.397	0.263	0.344	0.367	0.411	0.382	0.334	0.323	0.363	0.286	0.000		
SEN12.02	0.364	0.221	0.292	0.302	0.309	0.324	0.248	0.209	0.333	0.246	0.189	0.000	
SEN12.03	0.398	0.240	0.325	0.353	0.378	0.363	0.307	0.259	0.322	0.263	0.109	0.115	0.000

SEN: Senegal.

## Data Availability

The datasets used and analyzed during the current study are available within the manuscript.

## Supplementary Materials

Supplementary Materials Supplementary Table 1: list of selected polymorphic SSR markers for MPOB-Senegal oil palm germplasm. Supplementary Table 2: distribution of private, rare (bold), and unique alleles among the 26 MPOB-Senegal germplasms for 35 microsatellite loci. Supplementary Table 3: allele frequencies of private allele and rare allele (<0.10, bold) in 26 families of MPOB-Senegal using 36 SSR markers.
