# Supplementary material for: Genetic Diversity Assessment of MPOB-Senegal Oil Palm Germplasm Using Microsatellite Markers
Source: Biomed Res Int. 2021 May 4;2021:6620645. doi: 10.1155/2021/6620645 (PMC8116142; doi:10.1155/2021/6620645)
Supplement: Supplementary Materials — Supplementary Table 1: list of selected polymorphic SSR markers for MPOB-Senegal oil palm germplasm. Supplementary Table 2: distribution of private, rare (bold), and unique alleles among the 26 MPOB-Senegal germplasms for 35 microsatellite loci. Supplementary Table 3: allele frequencies of private allele and rare allele (<0.10, bold) in 26 families of MPOB-Senegal using 36 SSR markers. [file 6620645.f1.docx]

Supplementary Table 1: List of selected polymorphic SSR markers for MPOB-Senegal oil palm germplasm

| **No** | **Locus** | **Forward and** Reverse primer |  | **Ann. T.** | **Size(bp)** | **Ch** | **Repeat Motif** |
| --- | --- | --- | --- | --- | --- | --- | --- |
| 1 | sMg00093 | **F:**CAATAACCTGAAACCCCTCTAATC | **R:**ATCTCCATCACCCTCTTCTTCTC | 57 | 241 | 4 | (CT)6 (CT)18 |
| 2 | sEg00035 | **F:**TTATTGATTGATGCAAGATACAC | **R:**TTGATAAAATACAAGAGATAGCA | 52 | 170 | 1 | (AT)9 |
| 3 | sEg00151 | **F:**ATCACAACAGCAGCAGCATC | **R:**CGCATCAAGAAACATGGAGA | 57 | 219 | 6 | (CAG)8 |
| 4 | sMg00050 | **F:**ATGAGAACGACAAGTAAAGAGC | **R:**GCTTAGTTTATACCATACTCTCCA | 50 | 203 | 12 | (TA)17 |
| 5 | sMo00023 | **F:**TGACCCAATGATACCACCAC | **R:**GAGAACACAAACAGATGCACAC | 53 | 243 | 11 | (GA)16 |
| 6 | sEg00126 | **F:**CCGTCTCAAAAGCCCTAAAC | **R:**TTGTTGTCCCACTCCCTCTT | 52 | 170 | 5 | (CGC)7 |
| 7 | sEg00041 | **F:**ATTTGCATGTTTATGTGTCC | **R:**AAATCATCCTCCTATGTCTCTTT | 52/55 | 150 | 3 | (GA)23 |
| 8 | sEg00025 | **F:**GCTGAATGTGTATGAGCAGTT | **R:**GATTTACAGACAGGGCTTTT | 53 | 192 | 2 | (TTA)10 |
| 9 | sMg00055 | **F:**CCATTCCCACCAGCACTAAT | **R:**CAAGAACAACAAAGACAAAGG | 54 | 249 | 7 | (TCA)10 |
| 10 | sEg00066 | **F:**ACTGATGCAGGAAAGAGGAA | **R:**GAAGTACACAAGGTAAGTTCATAG | 52 | 200 | 16 | (AT)8 |
| 11 | sEg00036 | **F:**GGACCCTTTTTGTTACTGTTT | **R:**AGCCTACCACAACTTCCTTT | 52 | 173 | 9 | (AG)9 |
| 12 | sEg00092 | **F:**AACGTGAGAGCCATAGAGATAG | **R:**TAATAGAAACTAGACCCGACCA | 52 | 173 | 13 | (TATG)6 |
| 13 | sEg00189 | **F:**AACGTGAGAGCCATAGAGATAG | **R:**TAATAGAAACTAGACCCGACCA | 57 | 276 | 6 | (CT)9 |
| 14 | sMg00234 | **F:**TCGATCTTCTATTTCTTCTCCAC | **R:**CTGCGGTTTATTTGCTCATAG | 52 | 218 | 10 | (CT)23 |
| 15 | sMo00077 | **F:**TAGGACCAACTCGCATATCAG | **R:**AGCGTGACAGCATGTAGAAAA | 56 | 207 | 4 | (TC)11 |
| 16 | sMg00192 | **F:**TAGGACCAACTCGCATATCAG | **R:**AGCGTGACAGCATGTAGAAAA | 52 | 217 | 8 | (TA)17 |
| 17 | sMg00077 | **F:**GTCCCGACAAAACAGCATTTAT | **R:**CCATGACCTTAAACTGGCATC | 52 | 196 | 12 | (CT)7 (CA)13 |
| 18 | sEg00061 | **F:**CTGCAACTGCAAATGAGACA | **R:**TCCACCAGAGGAGGGTTAGT | 52 | 175 | 14 | (AG)9 |
| 19 | sMo00081 | **F:**TATAACCCCATGACGCATTCAC | **R:**AGCCGACTACATGCAAAAACC | 57 | 170 | 7 | (CT)18 |
| 20 | sMg00136 | F:GAATCCCACCAGTATCCATCTT | R:ATTTAGTATCCCCCTTCCCATT | 56 | 150-250 | 15 | (AG)11 |
| 21 | sMo00131 | F:GCATAGGAGCACTAATAACAACC | R:AGCCATGCAAGACAAGTATAGA | 54 | 200-300 | 15 | (TTA)19 |
| 22 | sMg00025 | F:GAGGAGGAGGGGAGAAGAGT | R:AAATACCATTCAGAGAAAGCAC | 52 | 198 | 16 | (TC)11 |
| 23 | sMo00053 | F:GATGTGGTGCCTCTTTTGTT | R:TGTGGTTAGTCTCATATTTGTCG | 54 | 212 | 9 | (TA)7(GA)7 |
| 24 | sMg00133 | F:CGCGAACAGAAAAAGAACAC | R:CGGAGTAGAGGAAACAAGATATACA | 55 | 194 | 5 | (TA)11 |
| 25 | sMg00027 | F:AAGACAGAATTGTTGTTTGACA | R:TTTGAGGGAAAAATGATAATAGA | 52 | 215 | 2 | (AG)12 |
| 26 | sMg00227 | F:TCTATTTCATCCAAATCTGCAC | R:TTTTCAGTTAGCGCATAGCAT | 54 | 229 | 1 | (CT)19 |
| 27 | sMg00205 | F:ACCATCAAAGTTCTCAAATGC | R:TTTGTGCGTGTATGTTTGTG | 52 | 200-212 | 3 | (CT)16 |
| 28 | sMo00121 | F:CAGGCAAGAGGTTGGTATTT | R:GATCAAAGTTCTTCACCCATTT | 54 | 198 | 9 | (TC)24 |
| 29 | sMg00147 | F:GTCGTAGGGTAGTCCGCTCAC | R:CTCACATCGCTTCTCCCATTAT | 56 | 206 | 8 | (AT)11 |
| 30 | mEgCIR0882 | F:CACGACGTTGTAAAACGACAAAGCGCGTAATCTCATAGT | R:TTGATCTTAGACATAACATACTGTA | 52 | 145 | 14 | (GA)8 |
| 31 | mEgCIR3546 | F:CACGACGTTGTAAAACGACGCCTATCCCCTGAACTATCT | R:TGCACATACCAGCAACAGAG | 52 | 286 | 11 | (GA)15 |
| 32 | mEgCIR3878 | F:CACGACGTTGTAAAACGACTAGTTTTCCCATCACAGAGT | R:ACAATATTTAGACCTTCCATGAG | 52 | 153 | 14 | (GA)25 |
| 33 | mEgCIR3282 | F:CACGACGTTGTAAAACGACGTAACAGCATCCACACTAAC | R:GCAGGACAGGAGTAATGAGT | 52 | 245 | 8 | (GA)20 |
| 34 | mEgCIR0369 | F:CACGACGTTGTAAAACGACGGGTAGCAAACCTTGTATTA | R:ACTTCCATTGTCTCATTATTCT | 52 | 206 | 14 | (GT)26 |
| 35 | sEg00009 | F:TCCACTGACAACAGGACTCA | R:AAAAACGCATCTCAGAGAGA | 52 | 182 | 10 | (AG)12 |

Supplementary Table 2: Distribution of private, rare (Bold) and unique alleles among the 26 MPOB-Senegal germplasm for 35 microsatellite loci

| **Family** | **Allele/Locus (Freq)** | **Allele/Locus (Freq)** | **Allele/Locus (Freq)** | **Allele/Locus (Freq)** | **Allele/Locus (Freq)** | **Allele/Locus (Freq)** | **Allele/Locus (Freq)** |
| --- | --- | --- | --- | --- | --- | --- | --- |
| SEN02.01 | **7/sEg00066 (0.071)** | - | - | - | - | - | - |
| SEN02.04 | 7/sEg00025 (0.1) | - | - | - | - | - | - |
| SEN02.05 | **1/sMg00093 (0.083)** | 12/sMg00205 (0.167) | 15/sMo00131 (0.1) | 19/sMo00053 (0.5) | - | - | - |
| SEN02.06 | 2/sMg00205 (0.1) | **4/sEg00035 (0.05)** | **7/sMg00192 (0.05)** | - | - | - | - |
| SEN02.09 | **2/sEg00126 (0.05)** | **2/sMg00025 (0.056)** | **12/sMg00050 (0.05)** | - | - | - | - |
| SEN03.06 | 3/mEgCIR3546 (0.143) | 3/sMo00081 (0.214) | **5/sEg00061 (0.071)** | **19/sMg00133** (0.071) | - | - | - |
| SEN03.07 | **1/sMo00081 (0.05)** | **2/sMo00023 (0.05)** | **5/mEgCIR3282 (0.05)** | 6/sMg00147 (0.15) | **13/mEgCIR3546 (0.05)** | 21/sMo00053 (0.143) | - |
| SEN04.01 | **10/sEg00036 (0.056)** | - | - | - | - | - | - |
| SEN04.03 | **1/sMg00227 (0.05)** | **2/sMg00227 (0.05)** | 7/sMg00050 (0.667) | 16/sMo00131 (0.2) | - | - | - |
| SEN05.01 | **1/mEgCIR0369 (0.05)** | **1/sEg00151 (0.056)** | 2/sMg00147 (0.111) | 7/mEgCIR3282 (0.111) | **8/mEgCIR0882 (0.056)** | - | - |
| SEN05.02 | 2/mEgCIR3282 (0.1) | 2/sMg00136 (0.1) | 2/sMg00192 (0.1) | 7/sMo00121 (0.1) | 9/sMg00205 (0.1) | 10/sMg00077 (0.25) | 13/mEgCIR3282 (0.1) |
| SEN05.03 | **2/sMo00121 (0.05)** | 5/sMg00205 (0.125) | **6/mEgCIR3282 (0.063)** | 7/sMg00133 (0.125) | 8/sEg00151 (0.222) | 8/sMg00025 (0.143) | **11/sMg00093 (0.056)** |
| SEN05.04 | 9/mEgCIR3546 (0.1) | - | - | - | - | - | - |
| SEN05.05 | 3/sEg00025 (0.25) | **3/sMg00133 (0.056)** | **7/mEgCIR3546 (0.071)** | **10/sMg00133 (0.056)** | - | - | - |
| SEN06.01 | **5/sMg00133 (0.071)** | - | - | - | - | - | - |
| SEN06.08 | **9/sMg00055 (0.05)** | **10/sEg00126 (0.05)** | **12/mEgCIR0882 (0.056)** | **13/sMg00093 (0.05)** | - | - | - |
| SEN07.03 | **1/sEg00066 (0.05)** | **8/sEg00009 (0.05)** | **9/sMg00136 (0.063)** | - | - | - | - |
| SEN07.05 | 1/sEg00025(0.143) | **5/sEg00126 (0.071)** | **7/sMo00053 (0.071)** | **8/sMo00053 (0.071)** | **9/sMo00053 (0.071)** | **15/mEgCIR3878 (0.071)** | - |
| SEN07.08 | **4/mEgCIR0369 (0.071)** | - | - | - | - | - | - |
| SEN10.03 | 2/mEgCIR0882 (0.333) | **2/sMg00093 (0.05)** | **4/sMo00053 (0.05)** | 6/sMg00077 (0.167) | **9/sMg00050 (0.071)** | 11/sMg00234 (0.25) | **21/sMg00133 (0.05)** |
| SEN10.05 | **3/mEgCIR0369 (0.05)** | 3/sEg00092 (0.111) | **4/sMg00205 (0.05)** | 6/sMo00077 (0.1) | 7/sMg00093 (0.111) | 10/sMg00050 (0.313) | - |
| SEN12.01 | 1/sMo00023 (0.5) | **6/sEg00061 (0.063)** | **6/sMg00025 (0.056)** | 11/sEg00126 (0.111) | **18/sMo00053 (0.056)** | - | - |
| SEN12.02 | **9/sMg00025 (0.071)** | - | - | - | - | - | - |
| SEN12.03 | 1/sMg00077 **(0.1)** | **4/sMg00077 (0.05)** | **10/sMo00081 (0.05)** | **12/mEgCIR3878 (0.05)** | **13/mEgCIR3878 (0.05)** | 14/mEgCIR0369 (0.15) | **15/mEgCIR0369 (0.05)** |

**Supplementary Table 3: Allele frequencies of private allele and rare allele (<0.10, Bold) in 26 families of MPOB-Senegal using 36 SSR markers**

|  | **AC** | **mEgCIR0369** | **AC** | **mEgCIR0882** | **AC** | **mEgCIR3282** | **AC** | **mEgCIR3546** | **AC** | **mEgCIR3878** | **AC** | **sEg00009** | **AC** | **sEg00025** | **AC** | **sEg00035** |
| --- | --- | --- | --- | --- | --- | --- | --- | --- | --- | --- | --- | --- | --- | --- | --- | --- |
| Private allele | 1 | **0.050** | 2 | 0.333 | 2 | 0.100 | 3 | 0.143 | 12 | **0.050** | 8 | **0.05** | 1 | 0.143 | 4 | **0.05** |
|  | 3 | **0.050** | 8 | **0.056** | 5 | **0.050** | 7 | **0.071** | 13 | **0.050** |  |  | 3 | 0.250 |  |  |
|  | 4 | **0.071** | 12 | **0.056** | 6 | **0.063** | 9 | 0.100 | 15 | **0.071** |  |  | 7 | 0.100 |  |  |
|  | 14 | 0.150 |  |  | 7 | 0.111 | 13 | **0.050** |  |  |  |  |  |  |  |  |
|  | 15 | **0.050** |  |  | 13 | 0.100 |  |  |  |  |  |  |  |  |  |  |
|  | **AC** | **sEg00036** | **AC** | **sEg00061** | **AC** | **sEg00066** | **AC** | **sEg00092** | **AC** | **sEg00126** | **AC** | **sEg00151** | **AC** | **sMg00025** | **AC** | **sMg00050** |
|  | 10 | **0.056** | 5 | **0.071** | 1 | **0.050** | 3 | 0.111 | 2 | **0.050** | 1 | 0**.056** | 2 | **0.056** | 7 | 0.667 |
|  |  |  | 6 | **0.063** | 7 | **0.071** |  |  | 5 | **0.071** | 8 | 0.222 | 6 | **0.056** | 9 | **0.071** |
|  |  |  |  |  |  |  |  |  | 10 | **0.050** |  |  | 8 | 0.143 | 10 | 0.313 |
|  |  |  |  |  |  |  |  |  | 11 | 0.111 |  |  | 9 | **0.071** | 12 | **0.050** |
|  | **AC** | **sMg00055** | **AC** | **sMg00077** | **AC** | **sMg00093** | **AC** | **sMg00133** | **AC** | **sMg00136** | **AC** | **sMg00147** | **AC** | **sMg00192** | **AC** | **sMg00205** |
| Rare allele | 9 | **0.050** | 1 | 0.100 | 1 | **0.083** | 3 | **0.056** | 2 | 0.100 | 2 | 0.111 | 2 | 0.100 | 2 | 0.100 |
|  |  |  | 4 | **0.050** | 2 | **0.050** | 5 | **0.071** | 9 | **0.063** | 6 | 0.150 | 7 | **0.050** | 4 | **0.050** |
|  |  |  | 6 | 0.167 | 7 | 0.111 | 7 | 0.125 |  |  |  |  | 13 | 0.111 | 5 | 0.125 |
|  |  |  | 10 | 0.250 | 11 | **0.056** | 10 | **0.056** |  |  |  |  |  |  | 9 | 0.100 |
|  |  |  |  |  | 13 | **0.050** | 19 | **0.071** |  |  |  |  |  |  |  |  |
|  | **AC** | **sMg00227** | **AC** | **sMg00234** | **AC** | **sMo00023** | **AC** | **sMo00053** | **AC** | **sMo00077** | **AC** | **sMo00081** | **AC** | **sMo00121** | **AC** | **sMo00131** |
|  | 1 | **0.050** | 11 | 0.250 | 1 | 0.500 | 4 | **0.050** | 6 | 0.100 | 1 | **0.050** | 2 | **0.050** | 15 | 0.100 |
|  | 2 | **0.050** |  |  | 2 | **0.050** | 7 | **0.071** |  |  | 3 | 0.214 | 7 | 0.100 | 16 | 0.200 |
|  |  |  |  |  |  |  | 8 | **0.071** |  |  | 10 | **0.050** |  |  |  |  |
|  |  |  |  |  |  |  | 9 | **0.071** |  |  |  |  |  |  |  |  |
|  |  |  |  |  |  |  | 18 | **0.056** |  |  |  |  |  |  |  |  |
|  |  |  |  |  |  |  | 19 | 0.500 |  |  |  |  |  |  |  |  |

Note: AC= Allele code
